# Stenting as a Rescue Treatment of a Pulmonary Artery False Aneurysm Caused by Swan-Ganz Catheterization

**DOI:** 10.1155/2014/893647

**Published:** 2014-12-28

**Authors:** Stefanie Keymel, Marc W. Merx, Tobias Zeus, Malte Kelm, Stephan Steiner

**Affiliations:** ^1^Division of Cardiology, Pneumology and Angiology, Department of Medicine, Medical Faculty, University Hospital Duesseldorf, 40225 Düsseldorf, Germany; ^2^Department of Cardiology, Angiology and Intensive Care Medicine, Robert Koch Hospital, 30989 Hannover, Germany; ^3^Division of Cardiology, Pneumology and Intensive Care Medicine, Department of Medicine, St. Vincenz Hospital, Auf dem Schafsberg, 65549 Limburg an der Lahn, Germany

## Abstract

Pulmonary vascular injury is a rare but life-threatening complication of Swan-Ganz catheterization. We report an 82-year old patient who underwent right heart catheterization by a balloon-tipped catheter because of suspected pulmonary hypertension. After deflation of the catheter in the wedge position, hemoptoe appeared associated with acute respiratory insufficiency requiring respiratory support by intubation and mechanical ventilation. Pulmonary angiography showed the formation of a false aneurysm of a segment artery of the left lower lobe. Immediate interventional therapy was performed by the implantation of two coated coronary stent grafts into the injured pulmonary artery thereby excluding the false aneurysm. Bleeding was stopped by this interventional approach while antegrade blood flow was maintained. Long term follow-up after 3 months showed an effective treatment with a completely thrombotic false aneurysm. However, despite oral anticoagulation and dual antiplatelet therapy, graft patency could not be achieved after 3 months. In summary, implantation of coated stents is a feasible and safe approach for the acute and long term treatment of potentially life-threatening condition of a pulmonary artery false aneurysm while treatment to achieve long term patency of the affected vessel still remains an issue to be resolved.

## 1. Introduction

Swan-Ganz catheter has been widely used for the diagnosis of pulmonary hypertension as well as hemodynamic monitoring in intensive care medicine or perioperative management for many decades. Although it seems to be a safe procedure, pulmonary artery injury occurs in 0.03%–0.47% [1–3]. Pulmonary artery injury is a severe, life-threatening complication with high mortality of more than 50% [2, 3]. Therefore, cardiologists and intensive care physicians should be aware of the symptoms of pulmonary artery injury and should define potential treatment strategies.

## 2. Case Report

**Figure 1:**
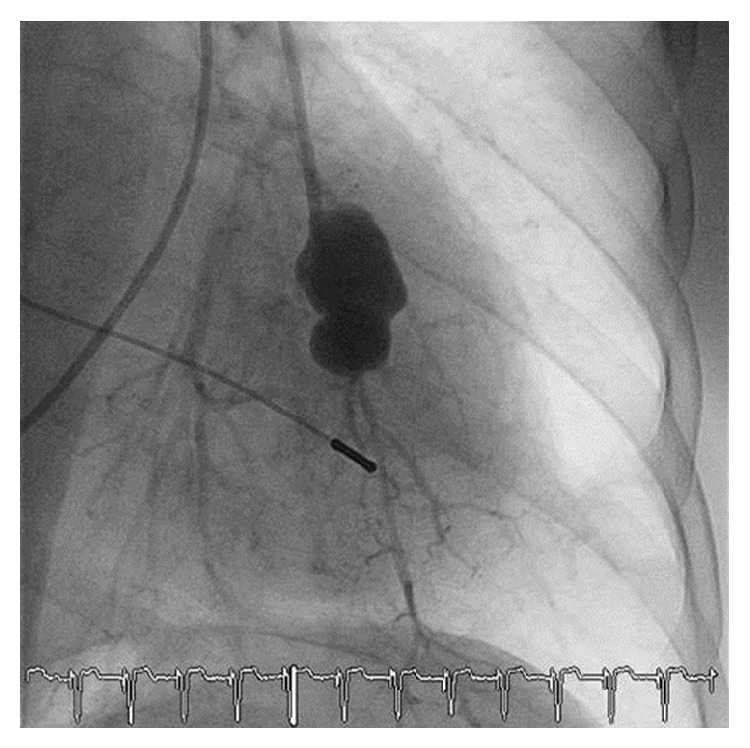
False aneurysm of a segment artery of the left lobe. Pulmonary angiography during hemoptysis presented extravasation of contrast media from a segment artery of the left lobe and the formation of a false aneurysm.

**Figure 2:**
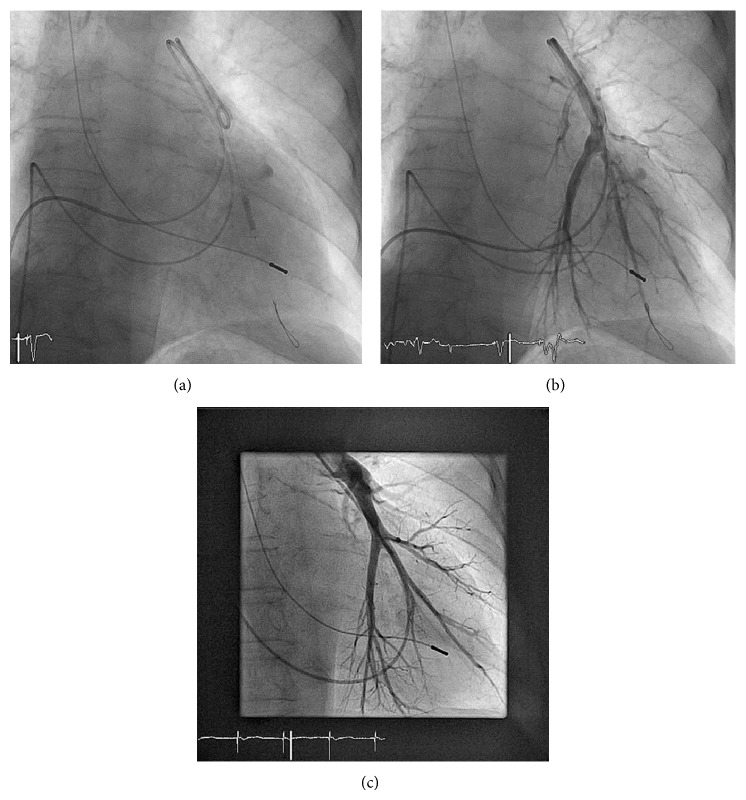
Stent graft implantation for the treatment of pulmonary artery false aneurysm. (a) False aneurysm was treated by the implantation of two coronary stent grafts to exclude the false aneurysm from the pulmonary artery. (b) Pulmonary angiography showed excellent result with unrestricted blood flow into the periphery while bleeding was stopped. (c) After 3 months there was a completely thrombotic false aneurysm without extravasation of contrast media. The pulmonary artery was obstructed at the level of the implanted stent grafts.

A female 82-year-old patient presented with instable angina and dyspnoea for further evaluation. Medical history included non-insulin-dependent diabetes mellitus type 2, arterial hypertension, permanent atrial fibrillation, and bradycardia-tachycardia syndrome (single chamber pacemaker implantation, 1989) as well as a stroke in 1995. Current medication was metformin, glimepiride, metoprolol, candesartan, simvastatin, hydrochlorothiazide, and pantoprazole, all once daily. Also, the patient was taking phenprocoumon for prevention of stroke due to atrial fibrillation. The INR on the day of the investigation was 1.8. Right heart catheterization was performed for the evaluation of pulmonary hypertension. Insertion of the Swan-Ganz catheter was uncomplicated. The measurement showed pulmonary hypertension with a prominent v-wave of 40 mmHg. Directly after deflation of the balloon the patient developed hemoptoe associated with respiratory insufficiency requiring intubation and mechanical ventilation, immediately. The balloon was reinflated immediately to prevent further bleeding. This manoeuver stabilizes hemoptoe; nevertheless, selective pulmonary angiography via Swan-Ganz catheter revealed a false aneurysm of a segment artery of the left lower lobe (Figure 1). Extravasation of contrast media could not be stopped by protamine to neutralize heparin. A pigtail catheter was inserted in the pulmonary artery of the left lower lobe to allow for displaying the culprit lesion using a J-wire. Accordingly we placed a 6 Fr multipurpose guiding catheter (LA5MB1 Launcher, Medtronic) and positioned a Balance Heavyweight guide wire (Abbott) distal to the aneurysm. Under angiographic control two coated stents of each 3.0 mm × 16 mm (GraftMaster Coronary Stent Graft System, Abbott) were placed in the pulmonary artery with excellent primary result (Figures 2(a) and 2(b)). Extravasation of contrast media was stopped and perfusion of the peripheral vascular bed was proven by angiography. No more hemoptysis was observed. Postinterventional pulmonary angiography two days after the intervention showed a residual false aneurysm. Therefore, a further stent graft (4.5 mm × 19 mm, GraftMaster Coronary Stent Graft System) was implanted proximal to the previously implanted stent grafts with complete separation of the false aneurysm from the feeding artery and unrestricted blood flow into the periphery. The patient was extubated two days after the initial event and discharged home 13 days after the event. Long term follow-up after 3 months by computed tomography and selective pulmonary angiography (Figure 2(c)) showed an effective treatment with a completely thrombotic false aneurysm without extravasation of contrast media. Despite oral anticoagulation and dual antiplatelet therapy (DAPT), the vessel was shown to be occluded in the 3-month follow-up without clinical symptoms of pulmonary embolism.

## 3. Discussion

We present a case report of successful acute and long term treatment of vascular injury leading to pulmonary hemorrhage and formation of a false aneurysm by endovascular stent graft placement. The risk of pulmonary artery rupture by Swan-Ganz catheterization has been shown to be determined by diverse factors including advanced age, female sex, comorbidities, and medication as well as the handling during catheterization (Table 1) [2–4]. In the literature, most of the ruptures occurred in elderly patients and in patients with pulmonary hypertension. This might be explained by remodeling and reduced vessel elasticity [4]. Further, a large pressure gradient across the balloon leads to wedging in a more peripheral position of the balloon. As critical medications, corticoids and oral anticoagulation have to be mentioned [5]. Regarding these risk factors, the patient in the reported case had to be considered as a high risk patient. In order to reduce the risk of catheter induced injury proper catheter placement and management are essential.

 How to reduce catheter induced pulmonary artery injury by proper catheter placement and management is as follows:
   inflating the balloon in a large proximal artery,
   “floating” the balloon-tipped catheter to its wedge position,
   minimizing the time in wedge position,
   avoiding excessive catheter manipulation or balloon hyperinflation,
   inflation of the catheter balloon with air, not fluids,
   deflating the balloon while traction is applied on the catheter.

Also, awareness of this complication is essential for the efficient management. The leading symptom is hemoptysis that may occur immediately after deflation of the catheter or during the later course due to rupture of a false aneurysm. Other symptoms include respiratory insufficiency potentially leading to asphyxia and hypovolemic shock. Asymptomatic pulmonary artery false aneurysm may be discovered by chest radiographs.

In view of the low incidence and the lack of controlled studies, there is no gold standard in treatment of pulmonary vascular rupture or false aneurysm. Treatment strategies vary between watchful waiting [6], surgery [4, 7], or angiographic embolization [5, 8]. Stent graft placement has been reported in one previous case report, only, which was also successful for the acute treatment of relevant hemoptysis due to Swan-Ganz catheter induced pulmonary bleeding [9]. In the reported case, there was no need for blood transfusion; this might be a consequence of early interventional therapy. The treatment of pulmonary artery rupture or pulmonary false aneurysm has to be tailored to the clinical condition of the patient as well as local conditions, for example, the availability of interventional prospects and skills.

In the reported case, acute pulmonary bleeding and hemoptysis with respiratory insufficiency were present immediately after deflation of the catheter in wedge position as the leading symptom or vessel rupture. Besides general measures such as securing hemodynamic and respiratory functions as well as optimizing coagulation, it is recommended to leave the catheter in position with slight inflation of the balloon in order to block antegrade blood flow to the injured vessel. In patients who survive the initial hemoptysis, the formation of false aneurysm has been reported in about 25% of the patients within minutes or even months [1, 10]. Although the formation of a false aneurysm prevents extravasation and further pulmonary bleeding, it might contribute to recurrent hemorrhage in 30–40% leading to death in 40–70% [6, 10]. In the reported case, interventional treatment by implantation of covered stents was a successful approach for the acute specific treatment of the pulmonary bleeding and the subsequent treatment of the remaining false aneurysm. Besides the acute treatment of the false aneurysm, stent implantation has been shown to be effective in the long term by complete exclusion of the aneurysm from the feeding artery after 3 months. Therefore, implantation of a stent graft should be considered as an alternative interventional approach for the treatment of a false pulmonary aneurysm.

Long term patency of the treated pulmonary vessels remains an issue to be resolved. In the previously presented case of stent implantation by Zuffi et al., long term follow-up after successful acute treatment of pulmonary hemorrhage has not been reported [9]. In the presented case, it was not possible to preserve perfusion of the pulmonary vessel in the long term follow-up despite DAPT and oral anticoagulation with phenprocoumon thereby accepting a substantially increased bleeding risk in an elderly patient with a history of life-threatening bleeding.

In summary, implantation of coated stent grafts is a feasible, safe, and effective therapy for the acute and long term treatment of pulmonary artery false aneurysm. This therapeutic approach might also be helpful in the interventional therapy of pulmonary bleeding due to pulmonary biopsy or pulmonary trauma.

## Figures and Tables

**Table 1:** Risk factors for pulmonary hemorrhage after Swan-Ganz catheterization [2–4].

Patient characteristics	Age >60 years
	Female sex

Comorbidities	Pulmonary hypertension
	Coagulation disorders

Medication	Systemic anticoagulation
	Long term steroid use

Others	Surgically induced hypothermia
	Cardiac decompression
	Cardiac manipulation during surgery
